# Early Postoperative Mortality Among US Veterans With a Robust Physiologic Reserve Undergoing Open or Endovascular Abdominal Aortic Aneurysm Repair

**DOI:** 10.1001/jamanetworkopen.2021.37245

**Published:** 2021-11-23

**Authors:** Katherine M. Reitz, Daniel E. Hall, Michel S. Makaroun, Edith Tzeng, Nathan L. Liang

**Affiliations:** 1 Department of Surgery, University of Pittsburgh, Pittsburgh, Pennsylvania; 2 Division of Vascular Surgery, University of Pittsburgh, Pittsburgh, Pennsylvania; 3 Veterans Affairs Pittsburgh Healthcare System, Pittsburgh, Pennsylvania; 4 UPMC, Presbyterian Hospital, Pittsburgh, Pennsylvania; 5 Wolff Center, UPMC, Pittsburgh, Pennsylvania; 6 Center for Health Equity Research and Promotion, Veterans Affairs, Pittsburgh, Pennsylvania

## Abstract

This cohort study uses data from the Veterans Affairs Surgical Quality Improvement Program database to examine the risk of early postoperative mortality among US veterans with a robust physiologic reserve undergoing open or endovascular abdominal aortic aneurysm repair.

## Introduction

The elective repair of abdominal aortic aneurysms (AAAs) mitigates their risk of AAA rupture. When the AAA anatomy meets instructions for use for an aortic endograft, endovascular aneurysm repair (EVAR) is preferred because of its lower rates of early postoperative complications and mortality compared with open surgical repair (OSR).^1^ This early EVAR advantage, however, must be weighed against the need for more frequent follow-up and increased aortic interventions.^2^

Traditionally, surgical procedures have been arbitrarily defined as high risk by a postoperative mortality rate of greater than 1%.^3^ However, early postoperative mortality is determined by both the physiologic stress of the intervention and the patient’s physiologic reserve (ie, robust or frail).^4^ Using data from the US Veterans Affairs Surgical Quality Improvement Program (VASQIP), we hypothesize that veterans with a robust physiologic reserve have equivalently low risks (≤1%) of early postoperative mortality after undergoing OSR or EVAR.

## Methods

This cohort study evaluated 2011 to 2019 data from the VASQIP database in such a manner that participants could not be identified. The study was thus deemed exempt from Veterans Affairs Pittsburgh Healthcare System Institutional Review Board approval and informed consent was waived. The study followed the Strengthening the Reporting of Observational Studies in Epidemiology (STROBE) reporting guideline.

We included all elective AAA repairs in the VASQIP database and excluded those missing a Risk Analysis Index (RAI) score. The RAI is a validated frailty measure, with scores indicating robust (≤20), normal (21-29), or frail (≥30) physiologic reserve.^4^ Our primary outcome was the 30-day mortality rate (reported with 95% CIs) stratified by frailty and age. Postestimation adjusted mortality rates were calculated from mixed-effects logistic regression including continuous variables (age, RAI) as fixed effects and categorical variables (year, hospital) as random effects, chosen a priori. Adjusted mortality equivalence was determined by a ±0.5% margin.^5^ Statistical analyses were performed with Stata 15.1 and GraphPad Prism 7.0 software.

## Results

**Figure 1.**
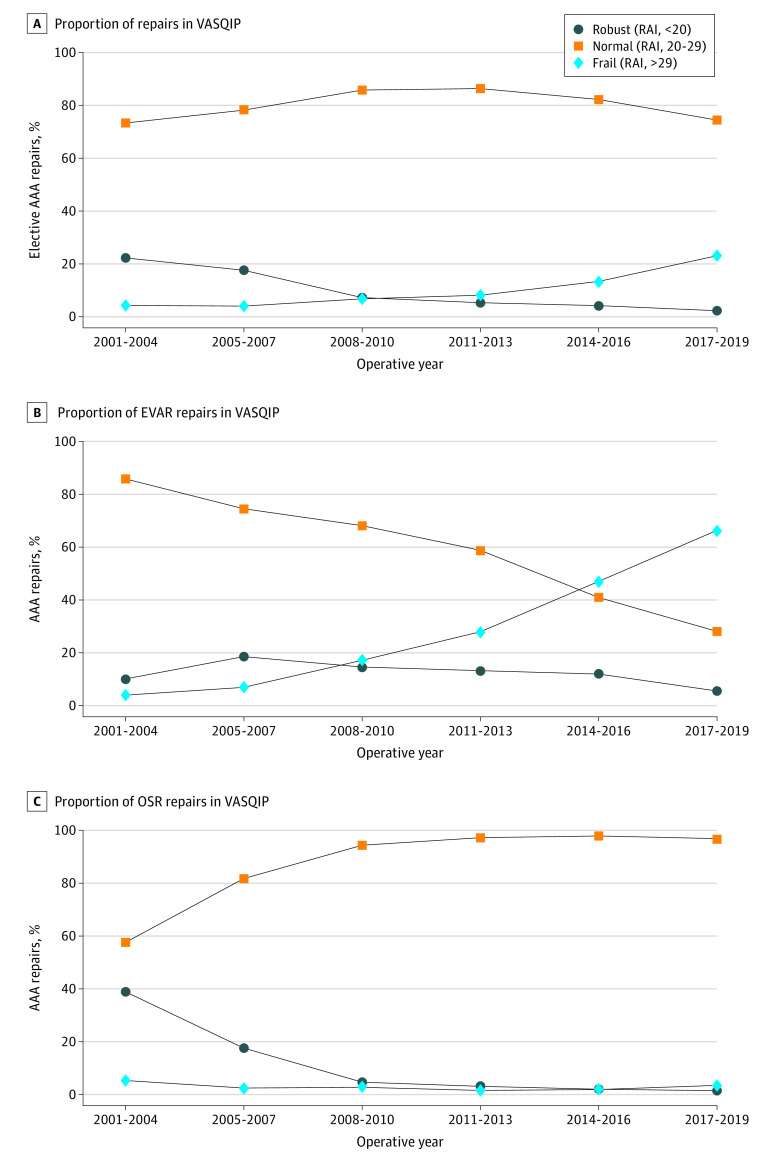
Proportion of Cases by RAI Measure of Frailty Line graphs show physiologic reserve levels of all patients undergoing elective AAA repair (A), among 28 975 patients (82.4%) undergoing elective EVAR (B), and among 6188 patients (17.6%) undergoing OSR (C) at 97 Veteran Affairs hospitals. AAA indicates abdominal aortic aneurysm; EVAR, endovascular aortic repair; OSR, open surgical repair; RAI, Risk Analysis Index; and VASQIP, Veterans Affairs Quality Improvement Program.

Of the 35 701 elective AAA repairs identified in the VASQIP database, we included 35 163 performed at 97 hospitals. Rates of EVAR (28 975 [82.4%]) increased among patients with a frail physiologic reserve (RAI ≥30; 4883 [16.9%]) over time (Figure 1). The mean (SD) patient age was 69.5 (7.5) years; 99% of veterans (34 914) self-identified as male, 91% (27 800) as White, and 3% (837) as Hispanic. Physiologic reserve was robust in 6.5% (2301), normal in 78.4% (27 552), and frail in 15.1% (5310). The observed 30-day mortality rates (95% CIs) were 0.61% (0.53%-0.71%) and 1.89% (1.57%-2.26%) for EVAR and OSR, respectively.

**Figure 2.**
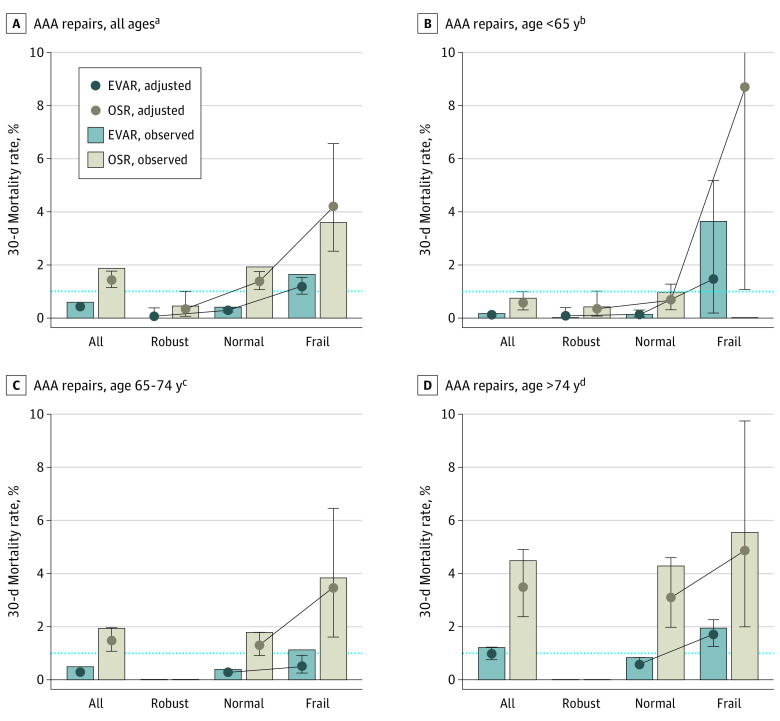
Observed and Risk-Adjusted 30-Day Postoperative Mortality After Elective Abdominal Aortic Aneurysm Repair, by Frailty and by Age and Frailty The observed (bars) and risk-adjusted (points) rates of 30-day mortality are shown for EVAR and OSR for all AAA repairs and stratified by frailty category (robust [RAI <20], normal [RAI 21-29; reference range], or frail [RAI >29]). Dashed horizontal lines indicate 1% postoperative 30-day mortality, and rates above this range indicate the historical definition of a high-risk procedure.^3^ All error bars represent 95% CIs. Equivalence between treatment groups was established for patients with robust physiologic reserve undergoing EVAR and OSR (A), as determined by an equivalence margin of ±0.5%, based on the more stringent 95% CIs than those previously reported (90% CIs).^5^ The adjusted 30-day mortality 95% CI was 1.1% to 28.0%. AAA indicates abdominal aortic aneurysm; EVAR, endovascular aortic repair; OSR, open surgical repair; and RAI, Risk Analysis Index. ^a^No. of patients: total, 35 163; EVAR, 28 975; OSR, 6188. ^b^No. of patients: total, 8343; EVAR, 6111; OSR, 2232. ^c^No. of patients: total, 18 446; EVAR, 15 379; OSR, 3067. ^d^No. of patients: total, 8374; EVAR, 7485; OSR, 889.

For the 2301 patients (6.5%) with a robust physiologic reserve undergoing EVAR (1429 [62.1%]) or OSR (872 [37.9%]), the observed (0.00% [0.00%-0.26%] vs 0.46% [0.12%-1.11%]) and adjusted (0.07% [0.00%-0.39%] vs 0.34% [0.07%-1.00%]) mortality rates were equivalently low at less than 1%, respectively (Figure 2A).

Among the 5882 patients (16.7%) younger than 65 years with a normal physiologic reserve who underwent EVAR (4545 [77.3%]) or OSR (1337 [22.7%]), the observed and adjusted mortality rates were also 1% or less. The observed mortality rates were 0.13% (0.05%-0.29%) and 0.97% (0.52%-1.66%) and adjusted mortality rates were 0.13% (0.05%-0.26%) and 0.67% (0.03%-1.27%) for EVAR and OSR, respectively (Figure 2B).

Among the 18 446 patients (52.5%) aged 65 to 74 years, higher postoperative observed and adjusted mortality rates (≥1%) were observed among the 3067 patients (16.6%) who underwent OSR (1.96% [1.50%-2.51%] and 1.47% [1.07%-1.96%]), respectively (Figure 2C). In addition, among the 8374 patients (23.8%) older than 74 years, higher postoperative observed and adjusted mortality rates were observed among the 7485 patients (89.4%) who underwent EVAR (1.23% [1.00%-1.51%] and 0.98% [0.77%-1.22%]), respectively (Figure 2D).

## Discussion

Overall, OSR was associated with greater early postoperative mortality compared with EVAR, which is likely attributable to the increased physiologic stress of OSR. However, both OSR and EVAR had equivalently low early mortality for patients with robust physiologic reserve. For frail patients, OSR was associated with higher early mortality, favoring EVAR where early mortality remained low. This finding may explain the increasing use of EVAR for frail veterans across the 18 years of VASQIP data included in our cohort study.

Among the oldest frail patients, even EVAR was associated with an early mortality rate of approximately 2%. This result illustrates how operative stress and patient frailty are independently and synergistically associated with increased early mortality.^4^ Therefore, age- and frailty-specific outcomes should inform decision-making regarding the method of AAA repair. Among patients with robust physiologic reserve who are anatomically eligible and have equivalent mortality for either EVAR or OSR, the shared decision-making process between surgeons and patients should include patient preferences regarding long-term surveillance after EVAR vs the greater surgical stress of OSR.^6^ Our findings are limited by the inclusion of only patients undergoing AAA repairs and by the lack of data on AAA anatomy, patient preferences, and other outcomes.

Among patients with robust physiologic reserve, OSR and EVAR were both associated with equivalently low rates of early postoperative mortality. Therefore, the repair choice for these patients should focus on eligible aortic anatomy and patient preferences for long-term follow-up.
